# Smart Elasto-Magneto-Electric (EME) Sensors for Stress Monitoring of Steel Cables: Design Theory and Experimental Validation

**DOI:** 10.3390/s140813644

**Published:** 2014-07-28

**Authors:** Ru Zhang, Yuanfeng Duan, Siu Wing Or, Yang Zhao

**Affiliations:** 1 College of Civil Engineering and Architecture, Zhejiang University, Hangzhou 310058, China; E-Mails: zhangru_306@163.com (R.Z.); ceyzhao@zju.edu.cn (Y.Z.); 2 Department of Electrical Engineering, The Hong Kong Polytechnic University, Kowloon, Hong Kong, China; 3 Department of Civil Engineering, Zhejiang University City College, Hangzhou 310015, China

**Keywords:** elasto-magnetic (EM), magneto-electric (ME), elasto-magneto-electric (EME) sensor, steel cable, stress monitoring

## Abstract

An elasto-magnetic (EM) and magneto-electric (ME) effect based elasto-magneto-electric (EME) sensor has been proposed recently by the authors for stress monitoring of steel cables with obvious superiorities over traditional elasto-magnetic sensors. For design optimization and engineering application of the EME sensor, the design theory is interpreted with a developed model taking into account the EM coupling effect and ME coupling effect. This model is able to approximate the magnetization changes that a steel structural component undergoes when subjected to excitation magnetic field and external stress, and to simulate the induced ME voltages of the ME sensing unit located in the magnetization area. A full-scale experiment is then carried out to verify the model and to calibrate the EME sensor as a non-destructive evaluation (NDE) tool to monitor the cable stress. The experimental results agree well with the simulation results using the developed model. The proposed EME sensor proves to be feasible for stress monitoring of steel cables with high sensitivity, fast response, and ease of installation.

## 1. Introduction

Steel cables are widely used in cable supported structures such as bridges and roofs. They play critical roles in ensuring the safety and performance of the structures. Stress monitoring of steel cables thus attracted much research interest in the nondestructive evaluation (NDE) and structural health monitoring communities [1–3]. However, real-time monitoring of cable stress for the in-service structures remains a challenging task. As such cables usually contain tens or hundreds of twisting wires or strands sheathed in a plastic protective cover or a duct filled by cement grout or grease, traditional stress monitoring method by using strain gauges is inapplicable or unable to measure the actual stress (not the relative variation of stress). The vibrating frequency method [3] is based on the relationship of cable tension and natural vibration frequency. However, its measurement accuracy is affected by many factors, for example, the bending rigidity and boundary condition of the cable, the installation of damping devices, and the effect of cable poly ethylene (PE) bushing. Microelectro mechanical systems (MEMS)-based pressure sensors [4,5] work on the principle that mechanical deformation of a thin diaphragm due to the pressure exerted on its contact surface is sensed by piezoresistive, capacitive, optical and resonant methods. They are widely used for measuring local pressures in the field of auto industry and medical care. Because the MEMS-based pressure sensors are essentially deformation/strain measurement based method, they cannot realize the monitoring of the actual stress measurement of the steel cables. There are also many difficulties for the current Metal Magnetic Memory (MMM) technology [6] to quantitatively measure the stress of steel cables because the weak detection signal is easily affected by the materials, residual magnetic field and external environment. By far, its application has not been seen in stress monitoring of steel cables. Elasto-magnetic sensors are promising for stress monitoring of steel cables because of their capabilities for actual stress measurement and noncontact monitoring [7–10]. Nevertheless, the low sensitivity, low signal-to-noise ratio, slow response, and complicated installation of the elasto-magnetic sensors due to the use of secondary coil as the detecting element limit their application flexibility. An elasto-magnetic (EM) and magneto-electric (ME) effect based elasto-magneto-electric (EME) sensor has been proposed recently by the authors for stress monitoring of steel cables with obvious superiorities over traditional elasto-magnetic sensors, such as high sensitivity, fast response, and ease of installation [11,12].

For better design and application of the EME sensor, a coupled model for numerical simulation taking into account the EM coupling effect and ME coupling effect is developed and verified in this article, which could fully explain its design rationality. A full-scale experiment with an engineering steel cable is carried out to verify the model and to calibrate the EME sensor. The EM coupling effect, also known as magnetomechanical effect, is the magnetization changes that a steel structural component undergoes when subjected to excitation field and external stress. Among the developed models such as the Jiles-Atherton (J-A) model, the Preisach model, and the homogenized energy model [13–21], the J-A model has succeeded in explaining many aspects of hysteresis and the magnetization process in ferromagnetic materials [22–25]. Therefore, the widely applied J-A model is adopted in the simulation of our EME sensor. For placement optimization of the ME sensing unit, the finite element analysis software ANSYS is used to simulate the magnetic field topography and to investigate the influence of model parameters on the magnetic field. The ME coupling effect [26–28] is an electric polarization response of an ME material subjected to an applied magnetic field. The ME sensing unit used here is made of ME laminated composites with superior ME effect due to the product effect of the piezoelectric effect and the magnetostrictive effect.

The ME voltage coefficient due to the ME coupling effect is obtained by the equivalent circuit method [28–31] and based on the previous work [11,12].

## 2. System Structure and Working Principle

**Figure 1.**
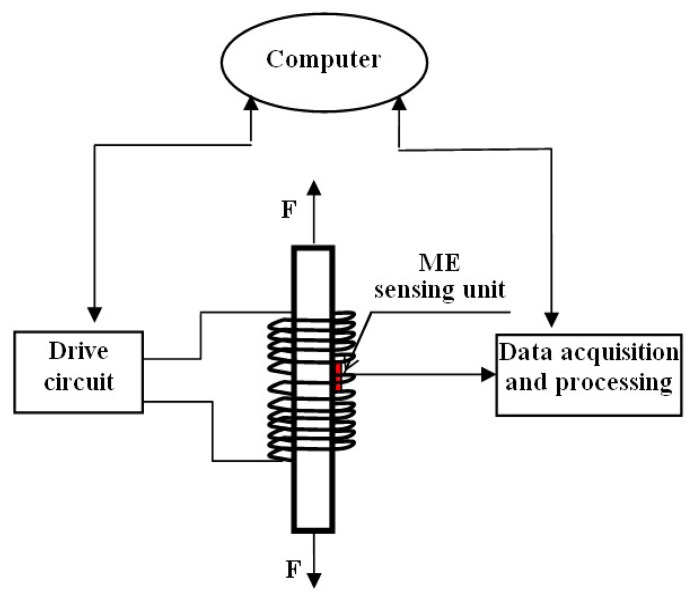
Schematic diagram of the proposed elasto-magneto-electric (EME) sensory system.

The EME sensor is mainly composed of a magnetic excitation part and a smart ME sensing unit, as shown in Figure 1. The magnetic excitation part, usually consisting of the magnetic coils and the drive circuit, provides the necessary magnetic field for the measured steel structural component and the surrounding area. The action of the stress on the steel member would result in changes in the magnetic properties (represented by magnetization intensity, permeability, *etc.*) of the ferromagnetic materials and thus in the distribution of magnetic field of the surrounding area, which is known as EM coupling effect. This effect mainly involves the conversion of the electromagnetic energy and mechanical energy. For the steel member under the combination of applied external stress *σ* and magnetic field *H*, the general equation of the system can be expressed as:

(1) $$B=\mu^{\sigma }H+D^{H}\Delta \sigma$$

where *B* is the magnetic induction, and $\mu^{\sigma }=(\frac{\partial B}{\partial H}){|}_{\sigma =\text{const}}$. Additionally, $\mu^{\sigma }{|}_{\sigma =0}$ becomes the normal magnetic permeability of the material. *D*^H^ is the EM coefficient, representing the changes of magnetic induction due to per unit stress σ under magnetic field *H*. So, the mechanical stress can be deduced if the magnetic induction *B* could be measured. The smart ME sensing unit converts the change of the magnetic induction into easily measured electrical signal represented by voltage, as the function of the ME coupling effect. Then, the stress can be reflected by the electrical signal output from the ME sensing unit.

## 3. Design Theory and Numerical Simulation

Steel cables are used as load-carrying members in a variety of civil structures, such as prestressing and post-tensioning tendons in prestressed concrete structures and stay cables in cable-stayed and suspension bridges. In this paper, the EM behavior of the material-steel cable PES(C)7-151 [32] is modeled. It is mainly composed of 151 high-strength steel wires of 7-mm-diameter sheathed in a PE protective cover. In this analysis, it is simplified into an ensemble with uniform permeability and conductivity along the longitudinal axis, since the magnetic field topography of the EME sensory system rather than the inside of the steel cable is the concern. The EME model is conducted by the following three subsections.

### 3.1. Simulation of the EM Effect

The application of the stress on the steel cable would result in the change of the magnetization *M*, namely EM coupling effect, which is modeled in the first subsection. The J-A mean field theory for ferromagnetic hysteresis is applied, which is applicable to both soft and hard magnetic materials. The coupled EM behavior of ferromagnetic materials is highly complex and we do not attempt to characterize all manifestations of the phenomenon in this paper. With the focus on changes in magnetization produced by the magnetic field and stresses, the present model is developed for isothermal behavior. The effect of stress on magnetization is quantified through a law of approach to the anhysteretic state.

In this model, the relationship of the applied stress σ, magnetic field *H*, and magnetization *M* can be obtained by the following steps:

(a) Under the combination of the applied σ and *H*, determined by minimization of a suitable thermo-dynamic potential, the effective magnetic field *H*_e_ has the form [16] of:
  (2) $$H_{\text{e}}=H+\alpha M+H_{\sigma }$$
  where α*M* is the Weiss interaction field responsible for the alignment of neighboring magnetic moments within domains. Furthermore, *H*_σ_ is the stress-dependent field component due to magnetoelastic interactions with the expression as:
  (3) $$H_{\sigma }=\frac{3}{2}\frac{\sigma }{\mu_{0}}\frac{d\lambda }{dM}$$
  where μ_0_ is the air permeability and λ *is* the magnetostrictive coefficient. Using the developed empirical model of the magnetostrictive coefficient λ and a Taylor series expansion of the stress-dependent part *r_i_*(σ) [13], *H*_σ_ is given by:
  (4) $$H_{\sigma }\frac{3\sigma }{\mu_{0}}\sum_{i=0}^{\infty }\left[iM^{2i-1}\sum_{n=0}^{\infty }\frac{\sigma^{n}}{n!}r_{i}^{\left(n\right)}\left(0\right)\right]$$
  So, when the terms up to *i* = 2, *n* = 1, *H*_e_ can be approximately written as:
  (5) He=H+αM+3σμ0{[r1(0)+σr1(0)′]M+2[r2(0)+σr2(0)′]M3}
(b) The anhysteretic magnetization *M*_an_(*H*, σ) at *H* and σ is identical to the anhysteretic *M*_an_(*H*_e_, 0) at field *H*_e_ and zero stress. In this model, by taking the first three orders in the Langevin function *L*(*z*), *M*_an_ is calculated by:
  (6) $$M_{\text{an}}=M_{s}(\frac{H_{\text{e}}}{3a}-\frac{H_{\text{e}}^{3}}{45a^{3}}+\frac{2H_{\text{e}}^{5}}{945a^{5}})$$
  where *M*_s_ is the saturation magnetization, *a* the parameter of the anhysteretic curve shape.
(c) The total magnetization *M* can be obtained by summing the irreversible magnetization *M*_irr_ and the reversible magnetization *M*_rev_:
  (7) $$M=M_{\text{irr}}+M_{\text{rev}}$$
  where *M*_irr_ is given by the law of approach to the anhysteretic state, in which the change rate of *M*_irr_ with respect to *W* is proportional to the difference between the irreversible magnetization and the anhysteretic magnetization:
  (8) $$\frac{dM_{\text{irr}}}{dW}=\frac{1}{\xi }\left(M_{\text{an}}-M_{\text{irr}}\right)$$
  where ξ is the reciprocal proportional coefficient; *W* is the elastic energy per unit volume indicating the influence of load type, and for the isotropic material with only uniaxial stress acting on it in the elastic stage can be given by:
  (9) $$W=\frac{\sigma^{2}}{2E}$$
  where *E* is Young's modulus.
  While, *M*_rev_ is related to the rotations of the domain walls, which can be expressed by:
  (10) $$M_{\text{rev}}=c\left(M_{\text{an}}-M_{\text{irr}}\right)$$
  where *c* is reversibility coefficient quantifying the linearized reversible component of magnetization owing to reversible domain wall movement.

**Figure 2.**
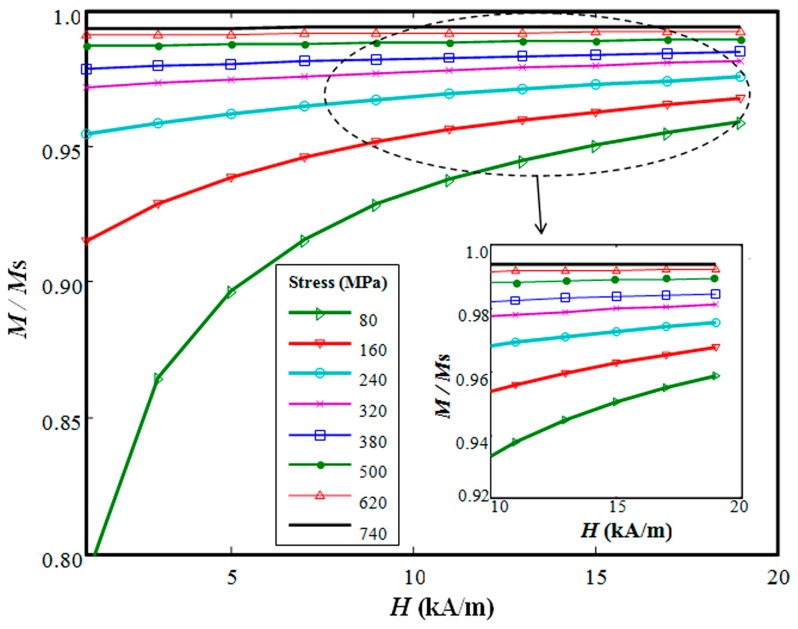
The simulated hysteresis loop of the descending part in the first quadrant under different stress levels obtained from solution of the model equations with *M*_S_ = 1.7 × 10^6^ A/m, *a* = 1000 A/m, *k* = 1300 A/m, *α* = 1.0 × 10^−3^, *c* = 0.1, *E* = 2.0 × 10^11^, *r*_1_(0) = 2 × 10^−18^, $r_{1}^{\prime }(0)=3\times 10^{-26}$, $r_{2}^{\prime }(0)=1\times 10^{-30}$, *r*_2_(0) = 5 × 10^−39^, ξ = 162 × 10^3^.

**Figure 3.**
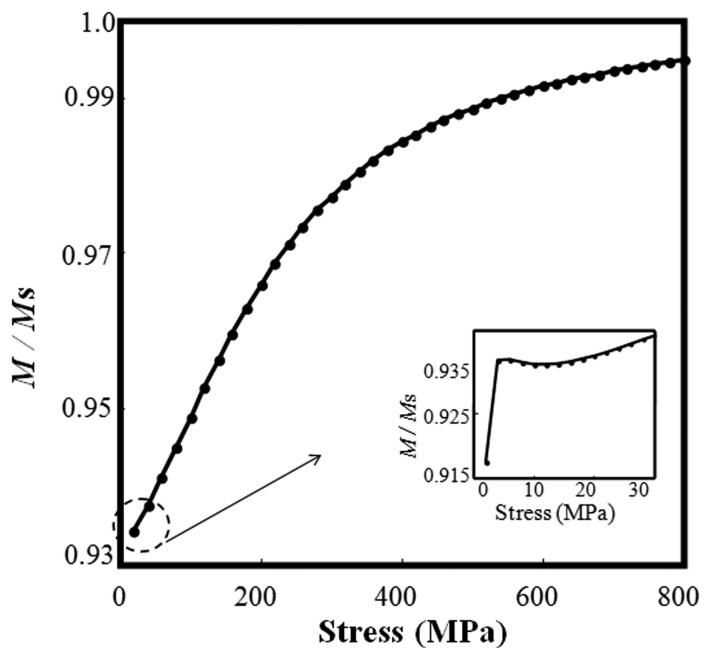
The simulated relationship of magnetization and stress with *H* = 15 kA/m.

This model can also be adopted for simulating other load types such as uniaxial stress, multiaxial stress, torsion, *etc.*, by adjusting the forms of *H*_e_ and *W* [33]. The anisotropic material can also be taken into account by modifying *H*_e_ [34]. For the case of steel cable discussed below, the above deduced equations are accurate enough. The equations have to be solved in numerical fashion because they involve a set of differential equations coupled with implicit nonlinear functions. The various types of magnetization therein were obtained using a Newton-Raphson scheme, and the final solution quantifies the nonlinearity. The hysteretic behavior of the ferromagnetic materials is calculated by a fourth-order Runge-Kutta scheme. The simulated hysteresis loop of the descending part in the first quadrant (this part is the concern for the EME sensor) under different stress levels is shown in Figure 2. It indicates that the higher the excitation *H* is, the better the linearity of the *M*–*H* relation becomes. However, the higher excitation *H* lowers the sensor sensitivity. Usually, in the nearly saturated magnetic field (approximately 14–18 kA/m as shown in Figure 2), good linearity of the *M*–*H* relation and good predictable repeatability can be obtained. The numerical solution of total magnetization–stress (*M*–σ) relation under constant *H* = 15 kA/m is shown in Figure 3 using the assumed parameters [10,13,15] of the tested steel cable, which is used as the constitutive relation of the iron core in the following subsection.

As is known, there exists the relationship between the magnetic induction *B* and the magnetic field strength *H* and magnetization *M* for the ferromagnetic materials:

(11) $$B=\mu_{0}\left(H+M\right)$$

From the calculated *M*–σ relation in Figure 3 and Equation (11), the EM coefficient can be obtained by:

(12) $$D^{\text{H}}=(\frac{\partial B}{\partial \sigma }){|}_{\text{H}=\text{const}}$$

It is seen from Figure 3 that the relationship of *M*/*M*_S_ and stress is irregular when the stress is smaller than 30 MPa as shown in the inset. This indicates that the effect of external stress on the domain wall motion has no advantage over that of the excitation magnetic field. In the larger stress stage, namely the stress larger than 30 MPa, it is noticeable that magnetization increases proportionally with the increasing external stress. This is due to the fact that the effect of external stress is much stronger than that of the excitation magnetic field on the change of magnetic properties, which can be explained from the view of magnetic domain theory [35]. The slope of the proportional relationship drops gradually with the stress increases, which may result from the increasing hindering of the domain wall motion. The relationship between the external stress and magnetic property is very useful in the non-destructive evaluation (NDE) of ferromagnetic materials.

## 3.2. Simulation of the Magnetic Field Topography

**Figure 4.**
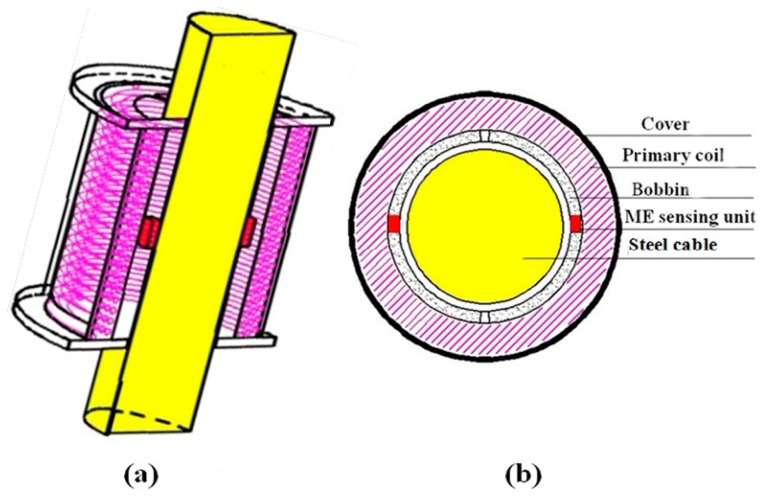
Structure of the EME sensor: **(a)** Longitudinal section; and (**b**) Cross-section.

**Figure 5.**
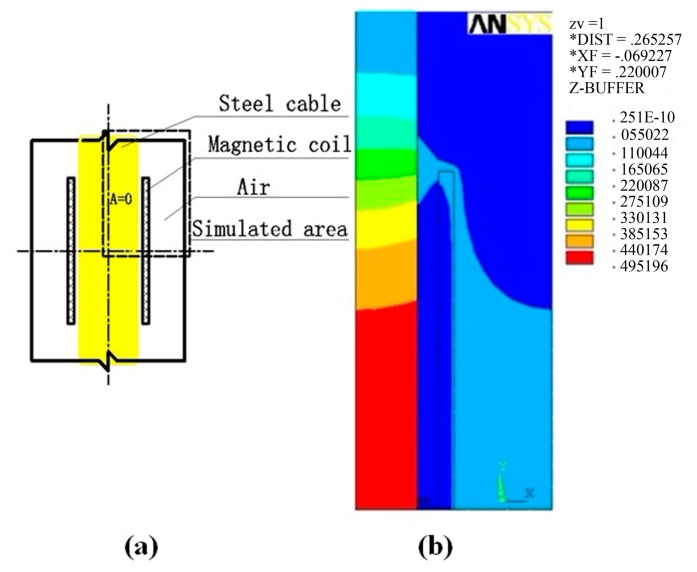
Finite element simulation: **(a)** Geometrical graph; and **(b)** The simulation result of one case. **A** denotes the magnetic vector potential.

From the working principle of EME sensory system, it is known that the change of the magnetic properties of the ferromagnetic materials is reflected by the output signal of ME sensing unit detecting the magnetic field at the surface rather than inside the material. So, the second subsection involves the characterization of the distribution of magnetic field under the known constant excitation field and external stress. As shown in Figures 4 and 5a, magnetic coil is wrapped around an iron core with some gap in between considering the coil skeleton. In this study, the magnetic coil of 2000 turns is wrapped around the steel cable, serving as the magnetic excitation part to generate the desired magnetic field.

In order to analyze the magnetic field of the EME sensory system, electromagnetic theory and the finite element method are necessary. This is done through magnetic field simulation and analysis using commercial finite element package ANSYS. The classical equation governing electromagnetic phenomena in terms of magnetic vector potential **A** in this system is [36,37]:

(13) $${\text{J}}_{0}+(-v\frac{\partial \text{A}}{\partial t})=-\frac{1}{\mu }\nabla \times \nabla \times \text{A}$$

where **J_0_** represents the input current density in the magnetic coil, and |**J_0_|** = *nI*_in_/*S*_c_ for the coil with the cross-section *S*_c_ and of *n* turns with a current of rms value *I*_in_. The second term in the left is eddy current density (namely the effect of demagnetizing field) in the magnetic core, where *ν* is the electrical conductivity. The right part is the total current density, where μ is the permeability.

Then, the magnetic flux density **B** and magnetic flux **Φ** in the coil can be respectively calculated by:

(14) B=∇×A

(15) $$\Phi =\iint_{S}\text{B}‥d\text{S}=\iint_{S}\nabla \times \text{A}‥d\text{S}=\oint_{C}\text{A}.d\text{I}$$

where ***C*** is the boundary of *S*.

Here, the estimated magnetic induction *B*_z_ along the *z*-axis (axial axis) is used as the main performance criterion for the magnetic coil in the finite element analysis. Using the decoupled constitutive relation from the first Subsection 3.1, two-dimensional (2-D) transient magnetic field simulation is practical and thus conducted. To minimize the complexity of the simulation, only a quarter of the domain is modeled due to symmetry and the corresponding boundary conditions are taken as denoted in Figure 5a. The air gap, steel cable and the skeleton are modeled with PLANE53 element. The magnetic coil is modeled by using PLANE53 with the circuit-coupled stranded coil option (KEYOPT (1) = 3). CIRCU124 elements are used for creating the circuit to generate the pulse current similar to that used in the experiment. The homogeneous Dirichlet boundary condition (**A** = 0) is specified along the vertical symmetry axis. The Neumann boundary condition ( $\frac{\partial \text{A}}{\partial n}=0$) governs the nodes along the horizontal symmetry axis. Transient magnetic field simulation is solved by full-wave method. When the current is passing through the magnetic coil, its dynamic magnetic induction *B*_z_ can be simulated.

Figure 5b shows a step of the representative simulated results under some stress using the derived μ*–H* relation of the steel cable. It is clearly found that the maximum magnetic intensity occurs in the axial center of the iron core. In our studied case, from analyzing the results of different nodes, the optimal position for placing the ME sensing unit is along the longitudinal direction and close to the outside surface of the iron core, which is the stress-sensitive magnetic field area with better stability. In addition, the magnetization simulation by using finite element analysis can also provide basis for illuminating the influence of the excitement frequency and the cable diameter on the magnetic field, and thus facilitating the implementation. It should be noted that this model is in fact a weak coupling model. For more precise finite element numerical results, the parameters of model and the analysis method [38–41] should be further studied. Anyhow, the thorough view of the magnetic field topography from the basic magnetic field analysis does enlighten the arrangement of the ME sensing unit and the design of the magnetizing energizer. The nodal values in two dimensional (2D)-case at each stress step can also be obtained for detailed design. The change of magnetic induction at the surface of the iron core has a approximate linear relationship with that inside the material, which can also be explained from the theory of demagnetizing field [42,43], and the transfer coefficient *K*_t_ is given by:

(16) $$K_{t}=\frac{\partial B_{\text{sur}}}{\partial B}$$

where *B*_sur_ and *B* are respectively the magnetic induction at the surface and inside of the iron core.

### 3.3. Simulation of the ME Effect

**Figure 6.**
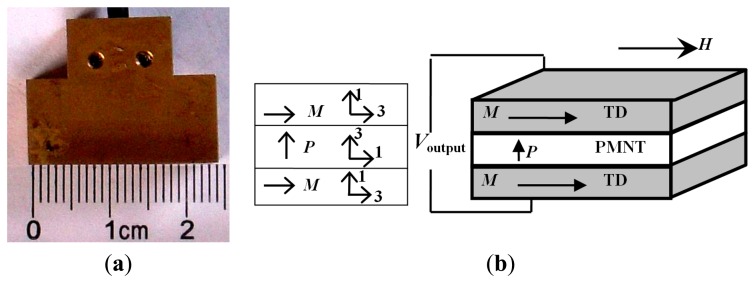
Schematic illustration of the ME sensing unit used in the EME sensor: **(a)** Photograph; and **(b)** Working principle, in which the arrows designate the directions of the magnetization (*M*) and polarization (*P*), respectively.

In the third subsection, the ME coupling effect of the ME sensing unit is investigated. The smart ME sensing unit is made of the Terfenol-D/PMN-PT/Terfenol-D (TD/PMNT/TD) laminated composites working in the longitudinal-transverse (L-T) mode, as illustrated in Figure 6. Owing to the great product effect of the piezoelectric effect and the magnetostrictive effect, it exhibited high magnetic field detection sensitivity, generally characterized by magnetic conversion coefficient α_v_. On the basis of equivalent circuit method, the developed *α*_v_ is determined [27,44,45] by:

(17) $$\alpha_{\text{v}}=\frac{dV_{3}}{dH_{3}}=\frac{n\left(1-n\right)t_{p}d_{33,\;m}d_{31,\;p}}{\varepsilon_{33}\left[n\left(1-k_{31,p}^{2}\right)s_{11}^{E}+\left(1-n\right)s_{33}^{H}\right]}$$

where *n* is a geometric thickness ratio of magnetostrictive layer to the total thickness of the laminate; *t*_p_ is the thickness of piezoelectric layer; *s*_11_*^E^* and *s*_33_*^H^* are the elastic compliances of the piezoelectric and magnetostrictive layers, respectively; *k*_31,_*_p_* and ε_33_*^T^* are electromechanical coupling coefficient and dielectric constant at constant stress of the piezoelectric material; *d*_33,_*_m_* and *d*_31,_*_p_* are the longitudinal piezomagnetic and transverse piezoelectric coefficients, respectively. The material parameters for Terfenol-D and PMN–PT single crystals are shown in Table 1 [27,44,45].

The magnetoelectric performances of the laminated composites are numerically calculated in References [44,45]. They exhibited good performance and the maximum ME voltage coefficient α_v_ is as high as 384 mV/Oe. The coefficient α_v_ is provided in Reference [45] as a function of applied ac magnetic field *H*_ac_ over the range of 10^−7^ T < *H*_bias_ <10^−3^ T for various DC bias magnetic strengths *H*_bias_ at the frequency of 1 kHz.

**Figure 7.**
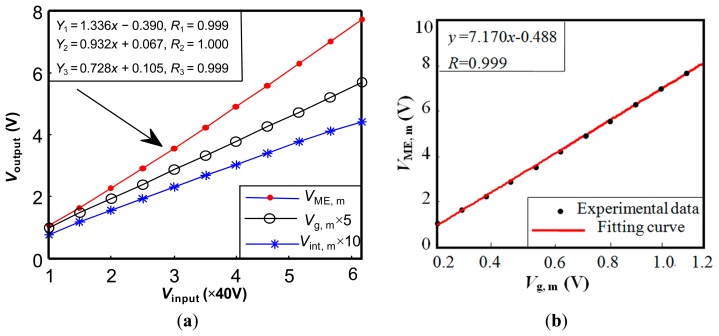
The performance tests of the ME sensing unit under pulse excitation: (**a**) The measured results by three methods under different input voltages *V*_input_; and (**b**) The relationship of *V*_ME_ and *V*_g, m_. *V*_ME, m_ is the peak value of *V*_ME_ output from the ME sensing unit. *V*_int, m_ represents the maximum value of the integral of the induced voltage output from a secondary coil. *V*_g, m_ denotes the peak value of the signal from a gaussmeter.

In the performance tests, the ME sensing unit displays great advantages such as high sensitivity, its self-powered nature, wide operational frequency range, fast response, and small size, *etc.* Its performance comparison with secondary coil and gaussmeter (PEX-045B, Litian Co. Ltd., Mianyang, China) under pulse excitation is also experimentally tested. Figure 7a plots their output signals under different input voltages. *V*_ME, m_ is the peak value of *V*_ME_ output from the ME sensing unit. *V*_int, m_ represents the peak value of the integration of the induced voltage output from the secondary coil. *V*_g, m_ is peak value of the signal from the gaussmeter. The good linearity among the three signals indicates that the ME sensing unit can be used to measure the magnetic induction. The relationship of *V*_ME, m_ and *V*_g, m_ is shown in Figure 7b. Compared with the hall probe connected to the gaussmeter, the ME sensing unit is of smaller size, more convenience, and higher sensitivity. Compared with the secondary coil, the ME sensing unit has a fast response (free of integration), higher sensitivity (tens of times of that using the secondary coil), small size and convenience of installation. The ME sensing unit is reliable and practical for measurement of magnetic characteristics.

The electrical signal *V*_ME_ output from the ME sensing unit at the selected position under the applied field and stress is obtained via the ME voltage coefficient. In practical applications, for given α_v_ and *V*_ME_, the change of magnetic induction *B*_sur_ can be obtained from:

(18) $$\Delta B_{\text{sur}}=\frac{1}{\alpha_{V}}\cdot \mu_{0}\Delta V_{\text{ME}}$$

### 3.4. Summary of the EME Model

From the above three subsections, it is clear that the EME sensor is directed to stress measurement of high sensitivity by detecting a change in spatial magnetic field Δ*B* corresponding to stress, which is implemented by the ME sensing unit possessing large ME effect. From Equations (12), (16), and (18), under a certain excitation magnetic field, the dependence of *V*_ME_ output from the EME sensor on the external stress can be deduced by the product effect of the EM effect and ME effect. Furthermore, the sensitivity of the EME sensor can be defined as:

(19) $$q_{\text{SMS}|H}=(\frac{\partial V_{\text{ME}}}{\partial \sigma }){|}_{H}=(\frac{\partial V_{\text{ME}}}{\partial B_{\text{sur}}}\cdot \frac{\partial B_{\text{sur}}}{\partial \sigma }){|}_{H}=(\frac{\alpha_{V}}{\mu_{0}}\cdot K_{t}\cdot D^{\text{H}}){|}_{H}$$

So, the stress can be obtained from:

(20) $$\sigma =Q_{\text{SMS}|H}\cdot V_{\text{ME}}+K_{0}$$

where $Q_{\text{SMS}|H}=(\frac{\partial \sigma }{\partial V_{\text{ME}}}){|}_{H}=K_{3}\cdot \frac{1}{q_{\text{SMS}|H}}$, and *K*_3_, *K*_0_ are the correction factors related to the selected variable and the relative position of the EME sensor and the tested member and other shifts.

**Figure 8.**
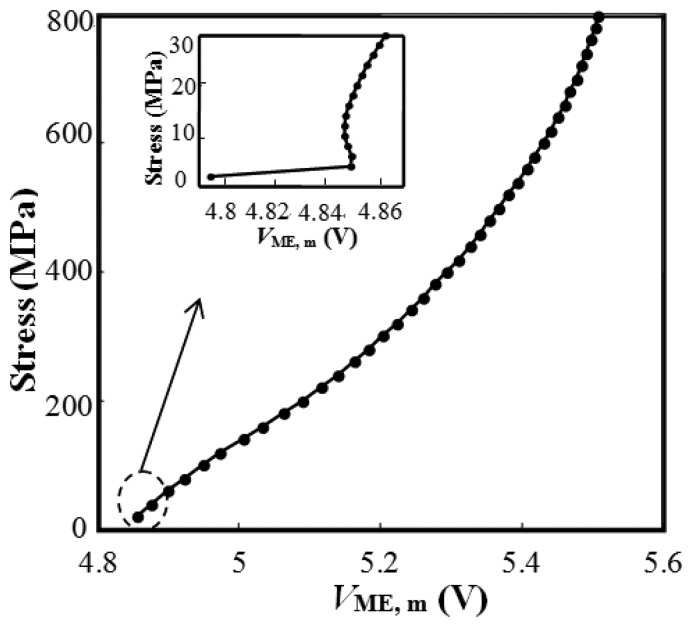
The simulated relationship of stress and *V*_ME, m_. *V*_ME, m_ is the max value of signal output from the ME sensing unit.

Figure 8 shows the relationship of stress and *V*_ME, m_, an approximate piece-wise linearity, which is consistent with magnetization-stress relationship as in Figure 3 with a coordinate interchange. Furthermore, the changed EM coefficient *D*^H^ of the modeled material directly leads to the piecewise linearity. The above model and numerical simulation indicate that the utilization of EM and ME effect based EME sensor is suitable for stress monitoring of steel components and that excellent performance can be achieved with proper design.

## 4. Experimental Validation

**Figure 9.**
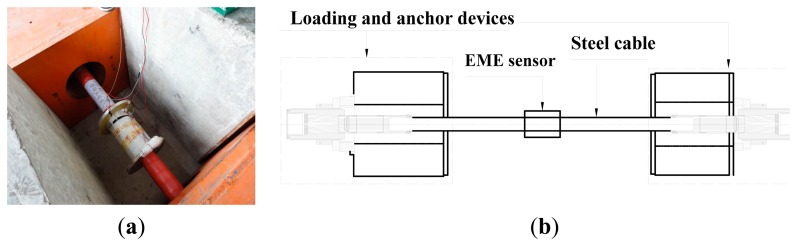
The setup of the full-scale experiment for the performance tests of the EME sensor: (**a**) Photo; and (**b**) Schematic diagram.

To test the capability and sensitivity of the proposed EME sensor and also to verify the developed model, several laboratory tests have been conducted [11,12]. Here, a full-scale experiment at room temperature was carried out on the steel cable PES(C)7-151. This cable was manufactured in conformity with the Chinese National Standard [32]. As shown in Figure 9, the hydraulic prestressing jack was used to stretch the steel cable to the necessary tensile load indicated by a load cell. The EME sensor was mounted at the middle of the steel cable. A drive circuit was used to control the current supply. The smart ME sensing unit measured the change of the magnetic field reflected by the output voltage *V*_ME_. Supported by the multifunction Data Acquisition (DAQ) device (USB-6211, NI), including D/A and A/D converters, both the drive circuit and ME sensing unit were controlled by the computer and all the inputs and outputs were displayed and processed in the computer with the software LabVIEW. The sampling frequency of 10 kHz is adopted in this experiment.

**Figure 10.**
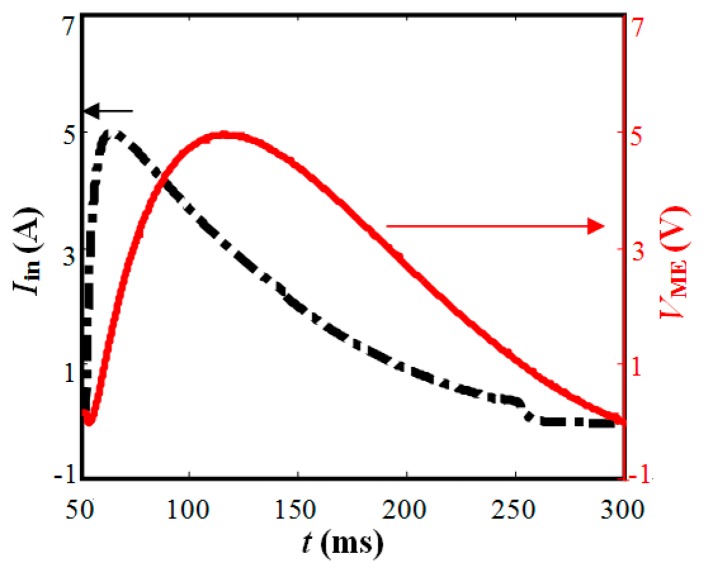
Typical acquired waveforms for conducting one test.

Figure 10 shows the typical output waveforms for conducting the tests. The input current *I*_in_ of the magnetic coil and the signals *V*_ME_ output from the ME sensing unit were recorded and processed by the data acquisition system. When the capacitors of the drive circuit discharge, a pulse current will be generated to pass through the excitation coil. The pulse current reaches the maximum level within a short time and then descends gradually. The changing current generates the magnetic field to magnetize the steel cable and the surrounding area. The action of the stress on the steel member would result in changes in the magnetic properties of the steel cable and thus in the distribution of magnetic field of the surrounding area. The magnetic induction changes of the surrounding area lag behind the electricity current changes. The smart ME sensing unit that is placed close to the steel cable converts the change of the magnetic induction into electrical signal and outputs voltage *V*_ME_ almost simultaneously. The peak of output signal *V*_ME_ lags behind the peak of the discharge current *I*_m_ because the magnetic induction changes of the surrounding area lag behind the electricity current changes.

**Figure 11.**
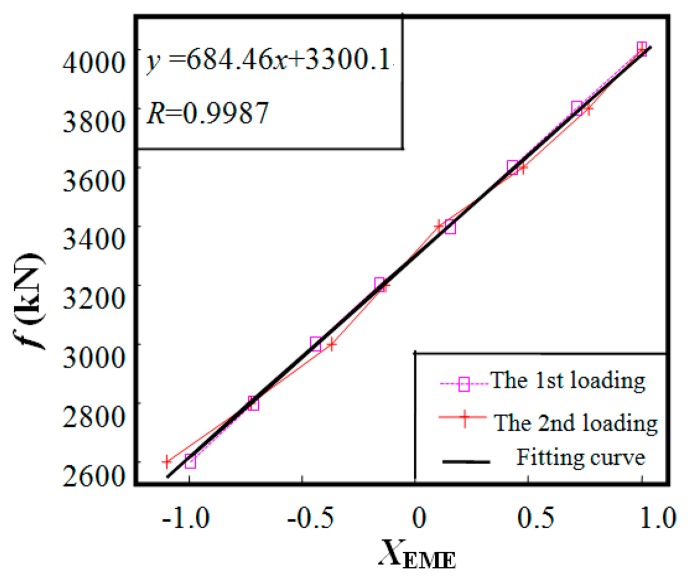
The results of the full-scale experiment constructed on the steel cable PES(C) 7-151.

The experimental result is shown in Figure 11. The ordinate load *f* is the loading value indicated by the load cell for large stress state (*f* = 2600–4000 kN, corresponding to the stresses of 447–688 MPa). The abscissa *X*_EME,_ signal output from EME sensor, is a normalized result of *V*_ME, m_ by setting the abscissa as 0, −1 and 1 for 3300 kN, 2600 kN, and 4000 kN, respectively. The results for the first and second loading tests are almost coincident, which proves the good repeatability of the EME sensor. As predicted by the numerical simulation in Figure 8, a good linearity between the output signal *X*_EME_ and the load *f* is observed. A linear regression equation by curve fitting from the two loading tests is obtained as *y* = 684.46 *x* + 3300.1 with the correlation coefficient *R* = 0.9987.

## 5. Conclusions

The design theory and experimental validation have been presented for the recently proposed EME sensor for stress monitoring of steel cables. The EME sensor is mainly composed of a magnetic excitation part and a smart ME sensing unit. The J-A mean field theory for ferromagnetic hysteresis is applied to simulate the EM effect and establish the relationship between the stress and the magnetic field in the measured steel cable. The magnetic field is simulated by the finite element method to relate the magnetic field at the location of the ME sensing unit with that of the measured steel cable. The ME effect of the laminated composites are characterized by the magnetic conversion coefficient determined by equivalent circuit method. Therefore, the relationship between the measured signal and the applied stress is established. A full-scale experiment is then carried out to verify the model and to calibrate the EME sensor to monitor the cable stress. The experimental results agree well with the simulation results using the developed model. The proposed EME sensor proves to be feasible for the in-service total-stress monitoring of steel structural members with high sensitivity, fast response, and ease of installation.

## Figures and Tables

**Table 1.** Material parameters for Terfenol-D and PMN–PT single crystals.

Materials	*d*_33,_*_m_* or *d*_33,_*_p_*	*d*_31,_*_m_* or *d*_31,_*_p_*	*s*_11_*^H^* or *s*_11_*^E^*	*s*_33_*^H^* or *s*_33_^E^	*k*_33_	*k*_31_	ε_33_*^T^*/ε_0_
**Terfenol-D**	1.2 × 10^−8^ Wb/N	−5.8 × 10^−8^ Wb/N	125 × 10^−12^ m^2^/N	40 × 10^−12^ m^2^/N	0.7	–	–
**PMN-PT**	2820 pC/N	−1126 pC/N	49.04 × 10^−12^ m^2^/N	119 × 10^−12^ m^2^/N	0.95	0.62	2134

## References

[1] Walther R. (1999). Cable Stayed Bridges.

[2] Virlogeux M. (2001). Bridges with multiple cable-stayed spans. Struct. Eng. Int..

[3] Kim B.H., Park T. (2007). Estimation of cable tension force using the frequency-based system identification method. J. Sound Vib..

[4] Bhat K.N. (2012). Silicon micromachined pressure sensors. J. Indian Inst. Sci..

[5] Liu X., Chen H., Huang Q.A., Young D.J. (2013). MEMS-based intraoperative monitoring system for improved safety in lumbar surgery. IEEE Sens. J..

[6] Dong L.H., Xu B.S., Dong S.Y., Chen Q.Z., Wang Y.Y., Zhang L., Wang D., Yin D.W. (2005). Metal magnetic memory testing for early damage assessment in ferromagnetic materials. J. Cent. South Univ. Technol..

[7] Sumitro S., Kurokawa S., Shimano K., Wang M.L. (2005). Monitoring based maintenance utilizing actual stress sensory technology. Smart Mater. Struct..

[8] Kvasnica B., Fabo P. (1996). Highly precise non-contact instrumentation for magnetic measurement of mechanical stress in low-carbon steel wires. Meas. Sci. Technol..

[9] Wang G.D., Wang M.L., Zhao Y., Chen Y., Sun B.N. Application of EM stress sensors in large steel cables.

[10] Wang G.D. (2006). The Application of Magnetoelasticity in Stress Monitoring. Ph.D. Thesis.

[11] Duan Y.F., Zhang R., Zhao Y., Or S.W., Fan K.Q., Tang Z.F. (2011). Smart elasto-magneto-electric (EME) sensors for stress monitoring of steel structures in railway infrastructures. J. Zhejiang Univ. Sci. A.

[12] Duan Y.F., Zhang R., Zhao Y., Or S.W., Fan K.Q., Tang Z.F. (2012). Steel stress monitoring sensors based on elasto-magnetic effect and using magneto-electric laminated composites. J. Appl. Phys..

[13] Jiles D.C. (1995). Theory of the magnetomechanical effect. J. Phys. D Appl. Phys..

[14] Jiles D.C. (1999). Erratum: Theory of the magnetomechanical effect. J. Phys. D Appl. Phys..

[15] Jiles D.C., Thoelke J.B., Devine M.K. (1992). Numerical determination of hysteresis parameters for the modeling of magnetic properties using the theory of ferromagnetic hysteresis. IEEE Trans. Magn..

[16] Sablik M.J., Jiles D.C. (1993). Coupled magnetoelastic theory of magnetic and magnetostrictive hysteresis. IEEE Trans. Magn..

[17] Dlala E. (2011). Efficient algorithms for the inclusion of the preisach hysteresis model in nonlinear finite-element methods. IEEE Trans. Magn..

[18] Smith R.C., Dapino M.J., Seelecke S. (2003). Free energy model for hysteresis in magnetostrictive transducers. J. Phys. D Appl. Phys..

[19] Naus H.W.L. (2011). Theoretical developments in magnetomechanics. IEEE Trans. Magn..

[20] Smith R.C., Dapino M.J. (2006). A homogenized energy model for the direct magnetomechanical effect. IEEE Trans. Magn..

[21] Umenei A.E., Melikhov Y., Jiles D.C. (2011). Analytic solution for variations of magnetic fields in closed circuits: Examination of deviations from the “standard” Ampere's law equation. IEEE Trans. Magn..

[22] Huang S.R., Chen H.T., Wu C.C., Guan C.Y. (2012). Distinguishing internal winding faults from inrush currents in power transformers using Jiles-Atherton model parameters based on correlation coefficient. IEEE Trans. Magn..

[23] Li W., Kim I.H., Jang S.M., Koh C.S. (2011). Hysteresis modeling for electrical steel sheets using improved vector Jiles-Atherton hysteresis model. IEEE Trans. Magn..

[24] Viana A., Rouve L.-L., Cauffet G., Coulomb J.-L. (2011). Analytical model for external induction variations of a ferromagnetic cylinder undergoing high mechanical stresses in a low magnetic field of any orientation. IEEE Trans. Magn..

[25] Chailloux T., Raulet M.-A., Martin C., Joubert C., Sixdenier F., Morel L. (2012). Magnetic behavior representation taking into account the temperature of a magnetic nanocrystalline material. IEEE Trans. Magn..

[26] Landau L.D., Lifshitz E.M. (1960). Electrodynamics of Continuous Media.

[27] Wang Y.J., Li M.H., Hasanyan D., Gao J.Q., Li J.F., Viehland D. (2012). Geometry-induced magnetoelectric effect enhancement and noise floor reduction in Metglas/piezofiber sensors. Appl. Phys. Lett..

[28] Yang F., Wen Y.M., Zheng M., Li P. (2006). Magnetoelectric response of magnetostrictive/piezoelectric/magnetostrictive laminate composite. Chin. J. Sens. Actuator.

[29] Yang F. Wen Y.M. Li P. Zheng M. Bian L.X. The resonant magnetoelectric response of magnetostrictive/piezoelectric laminated composite under the consideration of losses Acta Phys. Sin. 2007 56 3539 3545(In Chinese)

[30] Dong S.X., Li J.F., Viehland D. (2003). Longitudinal and transverse magnetoelectric coefficients of magnetostrictive/piezoelectric laminate composite: Theory. IEEE Trans. Ultrason. Ferroelectr. Freq. Control.

[31] Dong S.X., Zhai J.Y., Wang N.G., Bai F.M., Li J.F., Viehland D., Lograsso T.A. (2005). Fe–Ga/Pb(Mg_1/3_Nb_2/3_)O_3_–PbTiO_3_ magnetoelectric laminate composite. Appl. Phys. Lett..

[32] (2001). Technical Conditions for Hot-Extruding PE Protection High Strength Wire Cable of Cable-Stayed Bridge.

[33] Chen Y., Jiles D.C. (2000). The magnetomechanical effect under torsional stress in a Cobalt ferrite composite. IEEE Trans. Magn..

[34] Ramesh A., Jiles D.C., Roderick J.M. (1996). A model of anisotropic anhysteretic magnetization. IEEE Trans. Magn..

[35] Bozorth R.M. (1951). Ferromagnetism.

[36] Palanisamy R., Lord W. (1979). Finite element modeling of electromagnetic NDT phenomena. IEEE Trans. Magn..

[37] Tandon S.C., Chari M.V.K. (1981). Transient solution of the diffusion equation by the finite element method. J. Appl. Phys..

[38] Ren Z., Ionescu B., Besbes M., Razek A. (1995). Calculation of mechanical deformation of magnetic materials in electromagnetic devices. IEEE Trans. Magn..

[39] Srairi K., Féliachi M., Ren Z. (1995). Electromagnetic actuator behavior analysis using finite element and parametrization methods. IEEE Trans. Magn..

[40] Vandevelde L., Gyselinck J., Wulf M.A.C.D., Melkebeek J.A.A. (2004). Finite-element computation of the deformation of ferromagnetic material taking into account magnetic forces and magnetostriction. IEEE Trans. Magn..

[41] Vandevelde L., Hilgert T.G.D., Melkebeek J.A.A. (2004). Magnetostriction and magnetic forces in electric steel: Finite element computations and measurements. IEE Proc. Sci. Meas. Technol..

[42] Xu M.X., Xu M.Q., Li J.W., Ma S.S., Xing H.Y. (2012). Discuss on using Jiles-Atherton theory for charactering magnetic memory effect. J. Appl. Phys..

[43] Zan H.P. (2008). Researches on Demagnetizing Field Theory of Magnetic Materials. Master's Thesis.

[44] Jia Y.M., Luo H.S., Or S.W., Wang Y.J., Chan H.L.W. (2008). Magnetoelectric and converse magnetoelectric responses in Tb*_x_* Dy_1−_*_x_* Fe_2−_*_y_* alloy & Pb(Mg_1/3_Nb_2/3_)_(1−_*_x_*_)_Ti*_x_* O_3_ crystal laminated composites. Chin. Sci. Bull..

[45] Wang Y.J., Or S.W., Chan H.L.W., Zhao X.Y., Luo H.S. (2008). Enhanced magnetoelectric effects in longitudinal-transverse mode Terfenol-D/Pb(Mg_1/3_Nb_2/3_)O_3_–PbTiO_3_ laminate composites with optimal crystal cut. J. Appl. Phys..

